# Demonstration of Solar Cell on a Graphite Sheet with Carbon Diffusion Barrier Evaluation

**DOI:** 10.3390/molecules25040785

**Published:** 2020-02-12

**Authors:** Hyelim Cho, Jaeyeon Kim, Seran Park, Soobong Kim, Hyunjong Kim, Hoon-jung Oh, Dae-Hong Ko

**Affiliations:** 1 Department of Materials Science and Engineering, Yonsei University, 50, Yonsei-ro, Seoul 03722, Korea; pomi1019@yonsei.ac.kr (H.C.); jaeyeonkim@yonsei.ac.kr (J.K.); parksr@yonsei.ac.kr (S.P.); 2 New Power Plasma Inc., 49, Yusang-ro, Jeonju-si KS004, Jeollabuk-do 54852, Korea; sbkim@newpower.co.kr (S.K.); hjkim1@newpower.co.kr (H.K.); 3 BIT Micro Fab Research Center, Yonsei University, 50, Yonsei-ro, Seoul 03722, Korea; hi5hj@yonsei.ac.kr

**Keywords:** carbon substrate, carbon diffusion barrier, graphite sheet, a-Si solar cell, flexible device

## Abstract

An amorphous Si (a-Si) solar cell with a back reflector composed of zinc oxide (ZnO) and silver (Ag) is potentially the most plausible and flexible solar cell if a graphite sheet is used as the substrate. Graphite supplies lightness, conductivity and flexibility to devices. When a graphite sheet is used as the substrate, carbon can diffuse into the Ag layer in the subsequent p-i-n process at 200–400 °C. To prevent this, we added an oxide layer as a carbon diffusion barrier between the carbon substrate and the back reflector. For the carbon diffusion barrier, silicon oxide (SiO_2_) or tin oxide (SnO_x_) was used. We evaluated the thermal stability of the back reflector of a carbon substrate using secondary-ion mass spectrometry (SIMS) to analyze the carbon diffusion barrier material. We confirmed the deposition characteristics, reflectance and prevention of carbon diffusion with and without the barrier. Finally, the structures were incorporated into the solar cell and their performances compared. The results showed that the back reflectors that were connected to a carbon diffusion barrier presented better performance, and the reflector with an SnO_x_ layer presented the best performance.

## 1. Introduction

The interest in renewable energy sources is constantly increasing due to the concurrent increase in environmental issues [1]. Therefore, the research and market of solar cells, which use a representative clean energy, have continued to grow [2]. Devices are becoming smaller and lighter [3,4], and an emerging trend for devices is the adaptation for flexible appliances [5]. On this basis, we developed the idea of a solar cell integrated to a flexible device [6].

Materials that can potentially overcome the shortcomings of conventional solar cell substrates have been investigated. Silicon and glass, which are conventionally used as substrates for solar cells, are expensive and rigid. Moreover, the absence of electrical conductivity is a limitation to their application in solar cells [7]. In contrast, carbon materials are light, have high electrical conductivity, can be processed in various forms [8,9,10,11,12] and can also be flexible [13]; thus, a solar cell with a carbon substrate is lighter and more flexible [14]. The graphite sheet is one of the most popular commercial substrates among the flexible carbon materials [5,13]. In this work, an a-Si, which can easily be used with various substrates, was modeled as the final structure [15]. A back reflector which consisted of zinc oxide/silver (ZnO/Ag) was added to increase the light efficiency [16,17].

The high diffusivity of carbon at low temperatures (<800 °C) must be considered when it is used as a substrate [18,19]. There is a possibility that carbon could diffuse into the Ag layer, which is simultaneously used as a back reflector and back electrode, in the subsequent p-i-n formation process at 200–400 °C. If the carbon diffuses into the Ag layer, it may act as an impurity in the back electrode, thus degrading the current density (*J_c_*) [20,21]. To prevent the diffusion of carbon, a diffusion barrier was added. For the barrier material, two types of oxide were used—silicon oxide (SiO_2_), which is a typical dielectric oxide, and tin oxide (SnO_x_), a transparent conductive oxide (TCO) widely used in solar cells [2,17]. In summary, a graphite sheet (flexible carbon substrate) was used to produce a flexible solar cell device, and the functionality of the carbon diffusion barrier at the back structure of an a-Si solar cell—which included a carbon substrate and a back reflector—was investigated.

To apply an inexpensive graphite sheet as a solar cell substrate, the interface with the back reflector and the Ag/graphite sheet were annealed at 200–400 °C, which accounted for the chemical stability. The effects of the SiO_2_ and SnO_x_ layers between Ag/graphite were compared in terms of deposition characteristics, reflectivity and the chemical stability of Ag. Finally, the electrical properties of the a-Si solar cells were measured.

The aim of this research was to take a first step toward demonstrating that solar cells can be applied to flexible devices using graphite sheets as substrates when the carbon diffusion barrier is added. By extending their range to flexible devices, new possibilities for solar cell applications become clear.

## 2. Materials and Methods

### 2.1. Structure and Materials

In this study, a graphite sheet (EYGS-121803, Panasonic, Osaka, Japan) was used as the carbon substrate. For the back reflector layer, ZnO and Ag were used at a ratio that showed effective light trapping [22,23,24,25]. For the carbon diffusion barrier, an oxide layer of SiO_2_ or SnO_x_ was used.

Before the thin film deposition, the substrate was cleaned to remove any contamination by organic matter using acetone, isopropyl alcohol (IPA) and deionized (DI) water. First, the substrate was immersed in acetone for 5 min, then immersed in IPA for 3 min. Finally, it was rinsed for more than 1 min in DI water. The remaining DI water was then removed using a nitrogen (N_2_) blowing gun.

Each thin film was deposited using a magnetron sputter (KVS-T8860, Korea Vacuum limited, Daegu, Korea). The SiO_2_, SnO_x_ and ZnO thin films were deposited using a radio frequency (RF) power magnetron sputter, a controlled argon (Ar) flow of 5 sccm and constant 100-W RF power. The process temperature was held at 25 °C. The Ag thin film was deposited by direct current (DC) magnetron sputter, with constant process pressure of 3 mTorr, Ar flow of 22 sccm and substrate temperature of 25 °C.

When the carbon diffusion barrier was assembled, 100 nm of SiO_2_ or SnO_x_ was first deposited. The thickness of the back reflector included 350 nm of Ag and 100 nm of ZnO. These values represented the highest reflection efficiency according to previous experiments.

### 2.2. Thermal Stability of the Back Reflector on Graphite Sheet

After the structure was assembled, annealing was performed to create an environment similar to the p-i-n process for carbon diffusion evaluation. A drying furnace (WINFUS) was used for 1 h at 200, 300 and 400 °C in an N_2_ atmosphere.

Physical properties were observed using transmission electron microscopy (TEM, JEM 2100F, JEOL, Tokyo, Japan) and ultraviolet-visible spectroscopy (UV-vis, Cary5000, Agilent, Santa Clara, CA, USA). These analyses were performed with the samples before annealing. The annealed samples were used only for diffusion evaluation. Diffusion characteristics were determined using time-of-flight secondary-ion mass spectrometry (TOF-SIMS, TOF.SIMS-5, ION-TOF, Munster, Germany).

### 2.3. Solar Cell Fabrication

**Figure 1:**
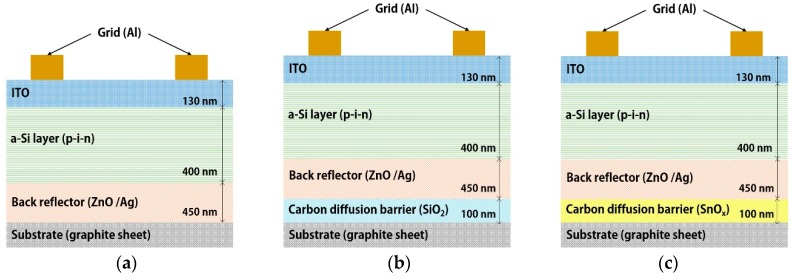
Structure of solar cells using graphite sheet: (**a**) without barrier layer, (**b**) with silicon oxide (SiO_2_) barrier layer and (**c**) with tin oxide (SnO_x_) barrier layer.

The electrical properties of the solar cell were measured by applying the graphite sheet as the substrate. The structures of the a-Si solar cells are shown in Figure 1. Aluminum (Al) grids and 130 nm indium tin oxide (ITO) constitute the top electrode, which is combined with a 400 nm p-i-n and a back structure. The back structure is the same as mentioned in Section 2.1, and all upper structures were deposited using plasma-enhanced chemical vapor deposition (PECVD). The upper structure was processed at 200−400 °C. First the n-layer and then the intrinsic layer were deposited at 300 mTorr in hydrogen (H_2_) and silane (SiH_4_) gas. In the p-layer, methane (CH_4_) and diborane (B_2_H_6_) gas were used in addition to H_2_ and SiH_4_ at 250 mTorr. Finally, the ITO layer was deposited in an Ar atmosphere at 300 mTorr. The solar cell performance was evaluated using the solar simulator under standard test conditions (STC = 1000 W/m^2^, 25 °C, AM1.5), which are the solar cell performance evaluation conditions specified by the International Electrotechnical Commission (IEC) [26,27].

## 3. Results and Discussion

### 3.1. Back Reflector Formed on Graphite Sheet

**Figure 2:**
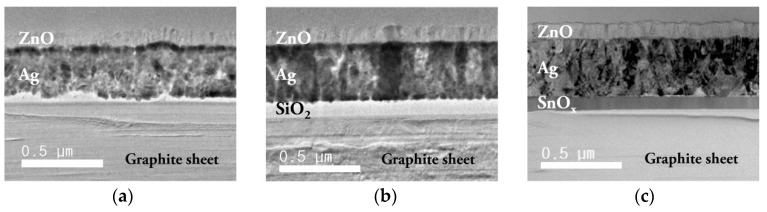
Cross-sectional transmission electron microscopy (TEM) images of the back reflector zinc oxide/silver (ZnO/Ag) on the graphite sheet deposited (**a**) without barrier layer, (**b**) with a 100 nm SiO_2_ barrier and (**c**) with a 100 nm SnO_x_ barrier.

Figure 2 shows the cross-sectional images of the multilayer samples. It can be confirmed that in the samples with a carbon diffusion barrier, the Ag thin film was more uniformly deposited. This phenomenon occurred because the carbon diffusion barrier reduced the influence of the graphite sheet roughness. 

**Figure 3:**
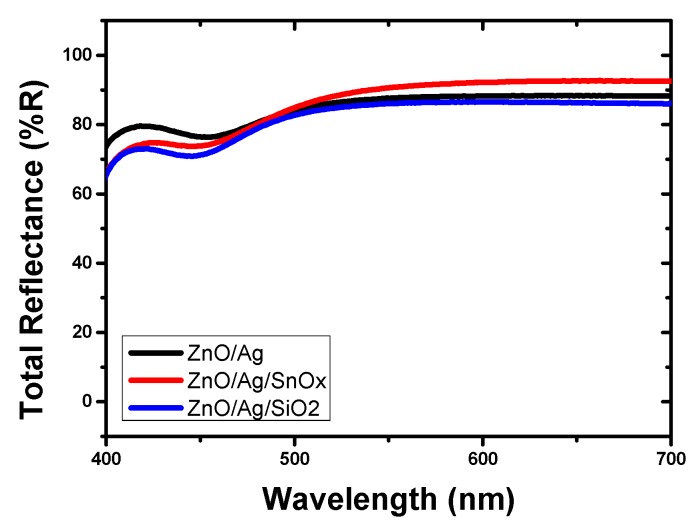
Total reflectance according to the back structure on the graphite sheet.

The reflection efficiencies of the samples with and without the carbon diffusion barrier were compared. Figure 3 demonstrates the reflectance of the back reflectors. The reflectance was measured in the visible light region, which is the wavelength range of amorphous solar cells [28].

Table 1 presents the average reflectance of each sample in the measuring range. The reflectance of the back reflector without carbon barrier (ZnO/Ag) was approximately 85.6%. When the SiO_2_ carbon diffusion barrier (ZnO/Ag/SiO_2_) was used, the reflectance was approximately 83%. For the SnO_x_ carbon diffusion barrier (ZnO/Ag/SnO_x_), the reflectance was approximately 87.7%.

The difference in the reflectance was not significant despite the carbon diffusion barrier. This result was attributed to the characteristics of the graphite sheet substrate, which was coated and presented a glossy surface. This feature appeared to attribute high reflectivity without a significant difference among the samples. Nevertheless, the reflectance was saturated at a higher value when the SnO_x_ carbon diffusion barrier was used.

### 3.2. Thermal Stability of the Back Reflector

We evaluated the carbon diffusion between the back reflector and the graphite sheet as the annealing temperature varied from 200 to 400 °C, which is the temperature range for the subsequent p-i-n formation process. The SIMS analysis was performed to compare the diffusion of carbon according to different barrier structures and annealing temperatures.

**Figure 4:**
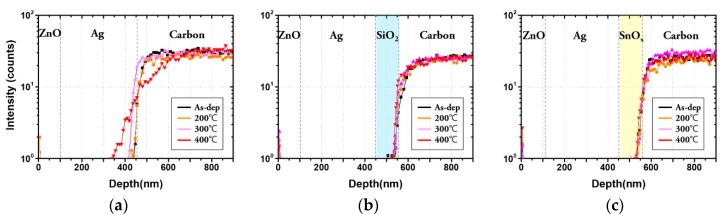
Diffusion of carbon by secondary-ion mass spectrometry (SIMS) in (**a**) ZnO/Ag/graphite sheet, (**b**) ZnO/Ag/SiO_2_/graphite sheet and (**c**) ZnO/Ag/SnO_x_/graphite sheet.

The carbon peak was extracted, and the results are shown in Figure 4. In the absence of the carbon diffusion barrier (Figure 4a), carbon was detected in the Ag layer even before annealing—that is, as-deposited. The diffusion decreased significantly in the presence of the barrier. Moreover, no carbon peaks were detected in the Ag layer when the barriers were used.

After annealing, each layer was diffused, regardless of the presence of a carbon diffusion barrier. Moreover, as the annealing temperature increased, the diffusion of carbon became more noticeable.

**Figure 5:**
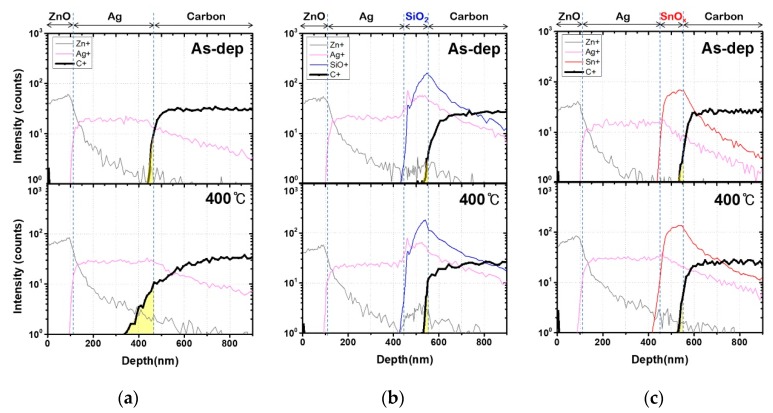
Secondary-ion mass spectrometry (SIMS) data before and after annealing at 400 °C with (**a**) ZnO/Ag/graphite sheet, (**b**) ZnO/Ag/SiO_2_/graphite sheet and (**c**) ZnO/Ag/SnO_x_/graphite sheet.

Figure 5 summarizes the SIMS depth profiles of the main components annealed at 400 °C compared to the values of the as-deposited samples. We focused on this case because it was the most critical temperature. We also compared the diffusion depth of carbon, which is shown in yellow on Figure 5.

For the samples with a carbon diffusion barrier (Figure 5b,c), the carbon diffused approximately 25 nm into the SiO_2_ and SnO_x_ layers. In the absence of the barrier (Figure 5a), carbon diffused approximately 113 nm into the Ag layer; therefore, carbon diffused better in the Ag layer than in the oxide layers. The results show that the barriers were effective in preventing carbon diffusion into the Ag layer.

When a carbon diffusion barrier is used, the structure becomes oxide/metal/oxide (OMO) [29,30]. This multilayer electrode structure is being actively researched for use in photoelectric devices [7]. Its advantages include low resistance and thermal and mechanical stability [30,31]. It also presents suitable characteristics for a flexible electrode [31]. The structure containing the carbon diffusion barrier is therefore beneficial not only to prevent carbon diffusion, but also to function as an effective electrode for solar cells.

The SIMS results show that the Ag peak saturates after the Ag layer (Figure 5a,c), but it is high only in the SiO_2_ thin film (Figure 5b). This result was attributed to the difference in density between SnO_x_ and SiO_2_ thin films. The X-ray reflectivity (XRR) analysis showed that the densities of SiO_2_ and SnO_x_ were 2.55 and 5.91 g/cm^3^, respectively. In addition, according to their molecular weights, the masses of SiO_2_ and SnO_x_ were 9.98 × 10^−23^ and 23.58 × 10^−23^ g, respectively. Thus, they have a molecular density close to 2.55 × 10^−22^/cm^3^. Consequently, the diffusion of Ag is more likely to occur in the SiO_2_ layer due to its lower molecular mass. In addition, when the deposition cross-section was examined, the SnO_x_ interface was cleaner than that of SiO_2_ and the influence of the interface was clear.

### 3.3. Amorphous Silicon Solar Cell Demonstration

**Figure 6:**
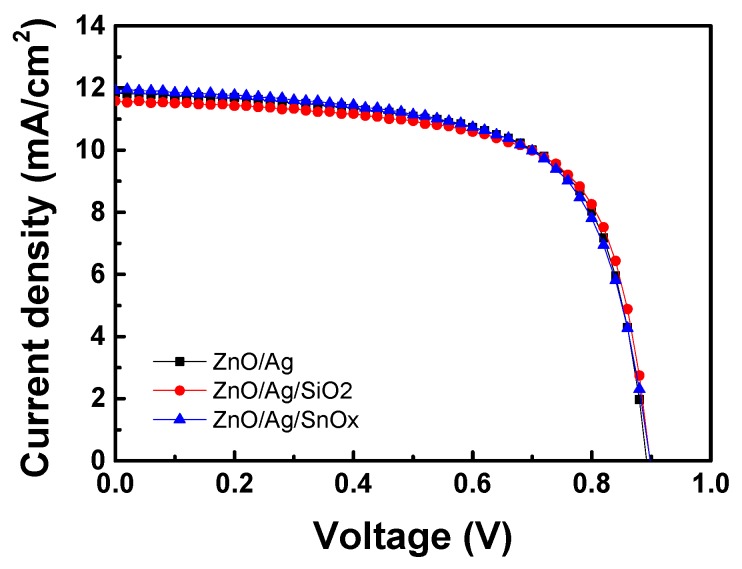
The current density depends on the back structure of the solar cell.

The solar cell was assembled so the feasibility of the carbon substrate and the barrier layer effect on the device characteristics could be examined. Due to the graphite sheet used as the substrate, the solar cells also showed flexibility by being able to bend. The performance of the solar cells is shown in Figure 6 and Table 2.

The performance of solar cells can be compared using conversion efficiency (*E_ff_*), which can be expressed by Equation (1). This equation shows that the ratio of incident light energy to electrical energy can be converted into electrical energy. Here, *P_input_* is the solar energy power incident on the solar cell, *P_output_* is the electrical energy output, fill factor (*FF*) is the charging factor, *I_max_* is the maximum current, *V_max_* is the maximum voltage, *I_sc_* is the short-circuit current and *V_oc_* is the open voltage when the resistance is flowing indefinitely.

(1) $$E_{ff}\;=\;\frac{P_{output}}{P_{input}}=\;\frac{I_{max}V_{max}}{P_{input}}=\;\frac{I_{sc}V_{oc}FF}{P_{input}}$$

The overall efficiency is not significantly lower than the efficiency of the a-Si solar cells described in previous papers, and all samples showed similar values [32]. Moreover, because the open voltage values were the same, it was concluded that the short-circuit current density (*J_sc_*) and *FF* affected the energy conversion efficiency. The reason for this result was attributed to the reduction of the influence of the lower part of the structure while the influence of the upper structure was applied.

## 4. Conclusions

In this study, graphite sheet was used as a substrate in solar cells for light and flexible devices. The effects of a carbon diffusion barrier on the backside of a solar cell where the back reflector was bonded to a carbon substrate were investigated. The thermal stability of the back reflectors on the graphite sheet was examined using SIMS with varying barrier layers and annealing temperatures up to 400 °C. We observed that the carbon diffused into Ag as the processing temperature increased; therefore, SiO_2_ or SnO_x_ were used as carbon diffusion barriers. Moreover, 350 nm Ag and 100 nm ZnO were combined in the back reflector layer to increase the efficiency on the back of the cell.

Three samples were prepared to compare the characteristics of the backs of the solar cells. The first one had only an as-deposited back reflector layer, while the second and third had an SiO_2_ or SnO_x_ carbon diffusion barrier.

When the carbon diffusion barriers were used, the film was deposited more evenly because the influence of the substrate was reduced. The reflection efficiency of the back reflector was maintained. It was possible to prevent carbon diffusion even when the structure of the back of the cell was only several nanometers thin. This experiment is a basic study that demonstrates the applicability of a graphite sheet to a solar cell. The practical use of the device would be possible by conducting further studies about the aging of the cell, such as temperature and light stability of the internal and external environments.

## Figures and Tables

**Table 1:** Average reflectance according to the back structure.

	Average Reflectance (%)		
Structure	ZnO/Ag	ZnO/Ag/SiO_2_	ZnO/Ag/SnO_x_
	85.6	82.9	87.7

**Table 2:** Electrical efficiency of the solar cell.

Back Structure	*V_oc_* (V)	*J_sc_* (mA/cm^2^)	*FF* (%)	*E_ff_* (%)
ZnO/Ag/graphite	0.89	11.84	66.79	7.05
ZnO/Ag/SiO_2_/graphite	0.89	11.57	68.01	7.07
ZnO/Ag/SnO_x_/graphite	0.89	11.91	65.42	7.00
